# Benefits of Mushroom-Based Supplements on Growth Performance, Immunocompetence, and Meat Quality in Poultry

**DOI:** 10.3390/ani14111517

**Published:** 2024-05-21

**Authors:** Safiu A. Suberu, Omoanghe S. Isikhuemhen, Tunde E. Ogundare, Deji A. Ekunseitan, Yewande O. Fasina

**Affiliations:** 1 Department of Animal Sciences, North Carolina Agricultural and Technical State University, Greensboro, NC 27411, USA; sasuberu@aggies.ncat.edu (S.A.S.); daekunseitan@ncat.edu (D.A.E.); 2 Department of Natural Resources and Environmental Design, North Carolina A&T State University, Greensboro, NC 27407, USA; omon@ncat.edu

**Keywords:** mushrooms, antibiotics, poultry, immunomodulatory, microbiota, additives

## Abstract

**Simple Summary:**

Simple Summary: The unrestricted usage of antibiotics led to the emergence of new antibiotic-resistant strains of pathogens, making it a concern in most countries. The restriction on the use of antibiotics in poultry has led to an increase in natural products that could serve as alternative antibiotics. Mushroom use in poultry nutrition is a major stride toward finding alternatives to antibiotics. This review aims to present a comprehensive overview of the role of different species of mushrooms commonly used in poultry in the performance, immunomodulatory actions, cholesterolemic properties, and meat quality of poultry birds.

**Abstract:**

The restriction on the use of antibiotics in poultry has led to an increase in the use of natural products that could serve as alternatives to antibiotics. Mushrooms contain bioactive compounds that exhibit antifungal, antiparasitic, antibacterial, antioxidant, antiviral, anti-inflammatory, and cytotoxic properties. Hence, they are being tested, revealing as performance-enhancing natural feed additives for livestock. This review focused on the role of different species of mushrooms commonly used in poultry on the performance, immunomodulatory actions, cholesterolemic properties, and meat quality of poultry birds. Different studies reviewed show that mushrooms could positively impact poultry production, improve growth performance, modulate immune response, exert tissue antioxidant activity, influence intestinal morphology, enhance gut microbiome, and improve lipid profile. The variations in their efficacy could be attributed to the variations in physicochemical properties of different species and dosage levels applied in the experiments. However, the use of mushrooms as a natural product supplement is in its infancy, and more basic, pilot and large-scale research is required to make it a viable approach for improving immune responses in the poultry industry.

## 1. Introduction

Subtherapeutic use of antibiotics has been a cornerstone of the poultry industry for the subclinical control of diseases, gut health maintenance, and growth promotion for several years [1]. The unrestricted usage of antibiotics led to the emergence of antibiotic-resistant strains of pathogens, making it a concern in most European countries, which led to a call for a ban on antibiotic use in animal production and a call for alternatives. Therefore, European Union countries have phased out the use of antibiotics at a subtherapeutic level in poultry rearing since 2006, leading to an increase in the use of natural products that could serve as alternative antibiotics. These alternatives, such as essential oils, plant extract oils, probiotics, postbiotics, and prebiotics, have been explored extensively as a possible in-feed antibiotic alternatives with satisfactory results [2]. Similarly, using medicinal mushrooms has become popular in the poultry industry due to their health benefits.

A mushroom is a fungus’s fleshy, spore-bearing fruiting body that is normally produced above ground. Typically, a mushroom has a cap (pileus), a stem (stipe), and gills (lamellae) or pores on the lower side of the cap. The microscopic spores produced by the pores or gills help the fungus spread across the ground or its occupant surface [3]. Mushrooms have nutritional and medicinal value, but in recent times, they have become more significant because of their nutraceutical value [4]. Several mushrooms have demonstrated bioactivity properties such as antifungal, antibacterial, antiparasitic, antiviral, antioxidant, anti-inflammatory, anti-proliferative, cytotoxic anticancer, antitumor, hepatoprotective, hypocholesterolemic, antidiabetic, anticoagulant, and anti-HIV properties [5]. An aqueous extract of *Cordyceps sinensis* and *Pleurotus australis* was reported to have antioxidant properties and inhibit the growth of *Bacillus subtilis* and *Streptococcus epidermidis* [6]. *Pleurotus ostreatus*, *P. tuberregium*, *Agaricus bisporus*, and *Ganoderma lucidum* have all been described to be more effective against bacteria such *as Staphylococcus aureus*, *Escherichia coli*, *Vibrio cholerae*, *Bacillus* spp. [7,8], and other fungi [9].

The beneficial properties of bioactive compounds in mushrooms show their potential as performance-enhancing natural feed additives in livestock [10]. Reports exist on the therapeutic and biological effects of mushroom use in poultry [5,6]. These studies indicate that mushrooms impact birds positively as they increase growth performance, modulate the immune response, improve gut health, exert tissue antioxidant activity, influence intestinal morphology, and improve lipid profile [1,2,4]. Also, it has been reported that the observed mushroom effects can be influenced by their origin, species, physiochemical composition, processing method, and level of supplementation [11].

There is clinical proof of significant medicinal properties in species such as *Agaricus bisporus*, *Agaricus blazei*, *Agaricus sylvaticus*, *Ganoderma lucidum*, *Lentinula edodes*, *Grifola frondosa*, *Hericium erinaceus*, and *Pleurotus ostreatus* in livestock. The extract of *Pleurotus ostreatus* in broiler’s water improved growth performance, dressed weight, and reduced the number of pathogenic bacteria in the gut [12,13]. Altop et al. [14] also observed improved feed efficiency with better growth performances and improved intestinal morphology in the broiler fed *Agaricus bisporus* stalk. The combinations of Shiitake, reishi, and cordyceps improved broilers’ body weight and boosted their immune response [15,16]. Shiitake inclusion in the layers’ diet was reported to improve egg production by reducing egg yolk cholesterol [17]. Likewise, the immunomodulatory effect of *Cordyceps militaris* was reported by Cheng et al. [18].

This paper attempts to review the role of different species of mushrooms commonly used in poultry on the performance, immunomodulatory actions, cholestrolemic properties, and meat quality of poultry birds.

## 2. Bioactive Components in Commonly Used Mushroom

Bioactive components are extra-nutritional constituents that are found in trace amounts in foods and offer pharmacological benefits beyond the intrinsic nutritional value of the product. Mushrooms have gained attention worldwide due to their functional bioactive components [19]. Mushrooms are considerably rich in compounds like polysaccharides, proteins, vitamins, etc. The presence of these compounds made them nutritionally valuable. For instance, the quality of protein found in mushrooms is reported to be better than most proteins present in plants [20]. They are regarded as a blend of different nutraceutical essential compounds, such as triterpenes, unsaturated fatty acids, peptides, alcohols, phenolic compounds, ergosterol, glycoproteins, etc. [21]. Similarly, the presence of compounds like glutathione, vitamin D, ergothioneine, and selenium in mushrooms confer antioxidant properties to them. Mushrooms are also known to possess antibacterial, antifungal, antiviral, anti-inflammatory, antidiabetic, antitumor, antithrombotic, hypolipidemic, hepatoprotective, and hypotensive activities [6,7,8,18,22].

Bioactive components in nutraceuticals are harmless substances but effective in promoting the growth performance and health of animals [23]. These substances could be polysaccharides, like β-glucans, from different mushrooms, e.g., Shiitake and Maitake, which are known to have medicinal properties [11]. The enormous diversities in the structure of natural compounds originating from mushrooms offer opportunities for drug discovery [24]. *Ganoderma lucidum*, for instance, contains polysaccharides, proteins, about 120 different triterpenes, and other bioactive molecules [5].

Bioactive compounds and their properties can be significantly influenced by the extraction methods. Most of the mushrooms have been extracted using methanol and ethanol, while the major compounds isolated are carbohydrates fucogalactan, β-glucan, and phenols constituents. Hu et al. [25] reported that ethanol extraction of *Inonotus obliquus* exhibited the strongest superoxide dismutases (SODs) like activity and anti-proliferative effect on colorectal adenocarcinoma cell line (DLD-1) compared with hot water extract, while freeze-drying was considered a good choice for polysaccharides preparation compared with hot and vacuum air drying and could be used to produce antioxidants for the food industry [26]. Glucans and specific proteins are responsible for most of the biological effects of mushrooms, such as immunomodulatory (pro-inflammatory or anti-inflammatory) and antitumor [27]. Glucan can exist in free form (β-glucan) or conjugated with protein (β-glucan + proteins), but the conjugated ones have shown greater immunopotentiation activity than the free glucans [5]. *Agaricus* sp. contains fucogalactan, polysaccharides, phenols and monosaccharides, and powerful antioxidants and is also capable of inhibiting the colonization of pathogenic microbial flora in the intestines [1,28,29,30].

Furthermore, other bioactive components found in mushrooms include cordycepin, gallic acid, and ergothioneine. *Cordyceps militaris* are known to contain ascorbic acid, which is a good antioxidant [31], as well as cordycepin, glucose, D-mannitol, 3,4-Oisopropylidene-D-mannitol, which have antimicrobial and antitumor activities [18]. Cordycepin is a bioactive compound found in cordyceps and acts as a potent antioxidant, neutralizing harmful free radicals and reducing oxidative stress [18]. *Tricholosporum goniospermum* contains gallic acid, which has antioxidant [32] and antimicrobial activities by producing irreversible changes in the properties of the bacterial membrane that normally lead to the rupture of their cell membrane [33]. Mushrooms have been reported to contain the sulfur-containing amino acid known as ergothioneine [34]. Ergothioneine also refers to 2-mercapto-histidine trimethylbetaine and has potent antioxidant activities [35]. Fungi, certain yeasts, actinobacteria, and some other microorganisms are capable of its synthesis. The concentration of ergothioneine varies depending on the geographical region, species, parts of the mushroom, etc. [36]. As reported by Chen et al. [36], the concentration of ergothioneine found in *Pleurotus ostreatus* in Japan is lower (944.1 mg/kg) than that found in Korea (1829.4 mg/kg). Among the mushrooms native to Asia, oyster species and Shiitake have the highest concentration of ergothioneine, while of those more frequently seen outside Asia, *Boletus edulis* are regarded as the best source [35,36]. Similarly, ergothioneine is more concentrated in the fruiting bodies of mushrooms compared with that of Mycelia [36]. These bioactive components have been reported to play key roles in the antibacterial, antioxidant, anti-inflammatory, and antiviral functions exerted in poultry. The bioactive components of some of the mushrooms used in poultry studies and their functions are presented in Table 1.

## 3. Role of Mushrooms on Poultry Performance

Performance in poultry refers to the measurable outcomes related to the production and health of birds within a farming operation. Some of the performance indicators are feed efficiency, growth rates, feed conversion efficiency, egg production, body weight gain, mortality rates, overall health, etc. As there is growing concern over the prophylactic use of antibiotics to increase production performance, the focus is shifting toward finding effective alternatives. One of the effective alternatives in the poultry sector is supplementing the poultry diet with mushrooms. It has been reported that mushrooms have beneficial effects on the feed intake, feed conversion ratio, and growth performance of birds [41]. *Agaricus bisporus* (Shiitake) is one of the most used mushrooms in the poultry sector because of its richness in antioxidants, polyphenols, and polysaccharides [29]. The beneficial effect of Shiitake on weight gain and feed intake was reported by Kavyani et al. [29]. The dosage level used differs from one study to another, resulting in different efficacy exhibited. For instance, Giannenas et al. [28] observed an improvement in the body weight, weight gain, and weight-to-gain ratio of turkey poults fed Shiitake at 20 g/kg at 70 days of age but no changes in the poult fed at 10 g/kg inclusion level. Similarly, broilers (Ross308) receiving Shiitake supplemented basal diet at an inclusion level of 20 or 30 g/kg had higher body weights [14,29]. Another species, *Agaricus brasiliensis*, has been identified as a possible source of prebiotics for broiler diets [44]. Guimaraes et al. [45] observed the best performance in broilers (Ross308) that were fed *A. brasiliensis*-supplemented diets at an inclusion level of 1.6 g/kg.

*Ganoderma lucidum* is a mushroom that is popular in Japan, the USA, and China as a useful source of feed supplements and medicine to repress the growth rate of tumors [46,47,48,49,50,51,52]. It is one of the most investigated species of mushrooms in Asia, and it is highly prized as a supplementary dietary feed. Phytochemical analysis showed that *Ganoderma* spp. contained carbohydrates, sugars, steroids, cardiac glycosides, saponins, and resins [53,54]. In the study by Ogbe et al. [53], pullets fed with the Ganoderma-supplemented diets at inclusion levels of 2% and 1% recorded a dose-dependent increase in weight gain and improved feed efficiency in pullets.

*Pleurotus ostreatus* powder included at 20 g/kg in the basal diet of broilers increased the feed intake and feed efficiency at the starter phase with no differences observed in the internal organ weights, carcass yield, small intestine, and cecum length of broilers [2]. The positive effects of mushrooms on feed efficiency and body weight of birds could be a result of the presence of carbohydrates like chitin and glucan, which act as prebiotics in maintaining the microflora balance of the intestinal tract of chickens, resulting in a better use of nutrients from the diet [16,55]. Meanwhile, their conclusion indicated that oyster mushroom powder as a growth promoter improved the performance indices of chicks during the starter and grower phases, but they did not sustain this improvement until the slaughter age. Fard et al. [56] noted that the inclusion rate is critical in applying oyster mushrooms to birds’ diets. Feeding chickens oyster mushroom wastes at 1% inclusion improved some parameters of performance. The role of different species of mushrooms on birds’ performance is summarized in Table 2.

## 4. Effect of Mushrooms on Egg Quality and Production

The egg industry is one of the vital sectors in poultry that interests numerous investments across the globe. In most countries of the world, eggs are regarded as the healthiest and most inexpensive source of animal protein [73]. The egg is a nutraceutical product that supplies nutrients necessary for human health. A whole chicken egg has a high water content (approximately 75%) and consists of organic and inorganic components [74,75]. Eggs contain essential components like fatty acids, minerals, amino acids, and vitamins; thus, they are considered a complete food and occupy a unique place among consumers. Eggs have antioxidant, anti-inflammatory, anticancer, and immunomodulatory components, which can play a key role in supporting the human body by maintaining different biological processes and protecting human organs from many diseases [76]. Eggs are regarded as functional food [77,78] because of their nutritional and health benefits. Economically, the success of any laying flock hinges on the number and quality of eggs produced and sold [78].

Including mushrooms in the layers’ diet is believed to enhance the quality and improve egg production. Wang et al. [31] reported that feeding *Cordyceps millitaris* at the inclusion rate of 20.0 g/kg resulted in an increase in egg weight, white weight, and shell strength. It is known that enhancing calcium availability and better absorption in the gut of laying birds could be a good strategy for improving eggshell quality and minimizing unmarketable eggs from aged layers [79]. Mahfuz et al. [60] suggested that a positive impact on increasing calcium retention and calcium deposition in the eggshell of layers might be due to the high contents of calcium in *Flammulina velutipes.* Similarly, Willis et al. [80] also found that *L. edodes* in layers’ diet improved egg production. Wang et al. [81] noted that although the dietary supplementation of Shiitake mushrooms increased egg production of layers but egg weight, shape index, and yolk color were not affected. The addition of *Pleurotus ostreatus* flour at a 0.6% inclusion rate also improved the egg weight of the laying hens [82]. Mahfuz et al. [60] observed better laying performance in layers fed with 4% and 6% *Flammulina velutipes*, respectively.

The Haugh unit measures egg protein quality based on the height of its egg white (albumen). Haugh units were higher in the eggs of birds fed *Flammulina velutipes* at a 6% inclusion rate [83], and albumen height was increased at a 0.5% inclusion of Shiitake [84]. An increase in albumen height is good because the higher the height, the better the quality. Yolk color could influence consumer preference as they tend to prefer a darker yolk color [85]. Odekunle et al. [86] noted optimal egg production with darker yolk color in the layers that were fed oyster mushrooms during the mid-lay phase. Carotenoid is a substance that improves egg yolk color and improves consumer acceptance. Oyster mushrooms are known to contain β-carotene [87,88], which may have a positive effect on egg yolk. The quality of eggs consumed is very critical to consumers. An increment in egg quality will enable egg producers to sell at higher prices than low-quality eggs, and it has been reported that the inclusion of *Pleurotus ostreatus* in the layers’ diet helps to improve egg quality [86].

## 5. Lipolytic and Cholesterolemic Properties

Edible mushrooms have a hypocholesterolemic effect. It was reported that a high quantity of serum triglycerides (TG) and Low-Density Lipoprotein (LDL) are linked with a greater risk of metabolic disorders such as fatty liver and abdominal fat deposition in chickens [89]. Meat lipid content and composition can influence its dietetic properties; thus, meat higher in High-Density Lipoproteins (HDL) and lower in LDL and Very Low-Density Lipoproteins (VLDL) are considered nutritionally good for consumption without predisposition to disease conditions [12]. The rate-limiting enzyme in the synthesis of cholesterol is 3-hydroxy-3-methylglutaryl coenzyme A (HMG-CoA) reductase; oyster mushrooms are known to contain mevinolin, which may be involved in decreasing the activity of this enzyme [90,91]. There was a decrease in the cholesterol, triglycerides, and LDL of the Japanese quail-fed 2% oyster mushrooms in their diet [92]. Daneshmand et al. [69] reported that the inclusion of 2 g/kg oyster mushroom increased the concentration of HDL but had no significant effects on Ross 308 broiler’s cholesterol, triglycerides, LDL, and VLDL. An increase in the level of HDL and a decrease in LDL and VLDL was also observed in the broilers administered with 100 g/1 L water of oyster mushrooms [12]. Similarly, Mahfuz et al. [41] reported that a 2% dietary inclusion of *F. velutipes* positively decreased total cholesterol (TC) and HDL in broilers (Arbor Acre). Similarly, Hassan et al. [70] also reported that a broiler (Ross 308) fed a diet containing 1% *Pleurotus osteratus* stem waste decreased total cholesterol. *Agaricus bisporus* inclusion at 1.5% used by Shamsi et al. [57] also had the same effect on lipid profile as it decreased blood cholesterol, and no reduction was observed in HDL and LDL. The effect of different mushroom species on cholesterolemic properties in poultry is presented in Table 3.

## 6. Immunomodulatory Effects of Mushrooms

Antibody responses are commonly used as measures of the humoral immune status of birds. Mushrooms are known to contain glycosides, alkaloids, polysaccharides, organic acids, and other bioactive compounds that are responsible for upregulating immune responses [97]. Antibody titers against Newcastle Disease (and sheep red blood cells (SRBCs) can be used to assess immune responses against diseases. Numerous studies have shown that including mushrooms can stimulate birds’ immune response. Mahfuz et al. [60] observed that the inclusion of *Flammulina velutipes* improved the immune response of pullets by increasing the level of IgY, interleukin-6 (IL-6), interleukin-4 (IL-4), and Serum interleukin-2 (IL-2). They noted that the presence of β-glucan in the mushroom was responsible. *Cordyceps militaris* exerts its immunomodulatory effect and inhibits inflammation-related gene expression in response to vaccination and lipopolysaccharides stimulation in broilers [18]. Some of the antibody functions of mushrooms were also ascribed to the dosage. For instance, Hassan et al. [70] reported an increase in the concentrations of immunoglobulin parameters in broilers fed 1% *Pleurotus osteratus* stem waste compared with a 2% inclusion. A *Pleurotus* species, *P. osteratus* var. *florida* was also reported to possess immunostimulatory effects due to the presence of beta-glucan [98].

The transmission of various infectious diseases to animals takes place through the alimentary tract. Therefore, understanding the nature and functions of various cellular components of gut-associated lymphoid tissue (GALT) gives us a view of the type of immunological responses operated in the gastrointestinal tract (GIT). Lamina propria and intestinal intra-epithelial leukocytes (iIEL) are some of the components of gut immunity [99]. Muthusamy [98] reported that β-glucan from a *Pleurotus* species had a significant immunostimulatory effect in broiler birds as reflected in the immune–effector activities of epithelial leucocyte cells (iIEL).

The health state of animals can be assessed via indices such as performance, clinical signs, etc., or through an in-depth immunological assessment. Serum immunoglobulins, which are secreted by B cells, are the principal effective molecules and might decently resonate with the actual humoral immunity. Serum immunoglobulin (IgY) was observed to be higher in broilers fed *Flammulina velutipes*, and no differences were observed in immunoglobulin A (IgA) and immunoglobulin M (IgM) concentrations. Among the serum cytokine concentrations, interleukin-2 (IL-2), interleukin-4, and interleukin-6 (IL-6) were higher in birds fed 2% *F. velutipes*. However, tumor necrotic factor-α (TNF-α) was not affected by feeding them *F. velutipes* [41]. Since TNF-α is an inflammatory factor, this may emphasize the anti-inflammatory function of *F. velutipes*. The serum IgA and IgY concentrations increased in the birds fed the diets containing 200 and 500 mg *Ganoderma lucidum* sporoderm-broken /kg feed [61,63]. The effects of mushrooms on the immune response of birds are summarized in Table 4.

## 7. Antioxidant Effect of Mushrooms on the Liver and Spleen of Birds

The imbalance between pro-oxidants and antioxidants can result in oxidative stress in birds, damaging molecules like proteins, lipids, carbohydrates, and DNA. Various harmful reactive oxygen or nitrogen species such as peroxyl (ROO), hydroxyl (HO), hydrogen peroxide (H_2_O_2_), superoxide (O_2_^−^), peroxynitrite (ONOO^−^), etc., play major roles in many poultry diseases [100]. Animals have natural mechanisms of fighting oxidative stress by producing free radical scavengers. This natural defense can be aided by providing feed rich in antioxidant compounds such as vanillic acid, selenium, zinc, vitamin C, vitamin E, copper, ergosterol, lovastin, ergothioneine, etc. [101].

Increased production of reactive oxygen species (ROS), including H_2_O_2_ and -OH, can lead to attacks on biological molecules [102]. The primary component of ROS is H_2_O_2_ released from the mitochondria. The -OH is also an extremely reactive free radical that reacts rapidly with almost every type of molecule, including sugars, amino acids, phospholipids, DNA bases, and organic acids [103]. An overabundance of ROS can cause oxidative stress by increasing peroxidation of cell lipids and, therefore, destroying the cell membrane activity by reducing the fluidity of the membrane and changing the activity of membrane-bound enzymes and receptors [104,105]. Free radical molecule levels and lipid peroxidation are usually controlled by the antioxidant defense system in the body. It consists of enzymatic components, such as superoxide dismutase (SOD), catalase, and glutathione reductase, and non-enzymatic components, such as glutathione and vitamin E [106,107]. Thus, in oxidative stress, the defense system aids the upregulation of the gene (enzymatic) expression and the production of non-enzymatic antioxidants [108].

There are examples of studies regarding the in vitro antioxidant activity of mushrooms with their phenolic contents [109,110], and their beneficial role in the poultry industry is also gaining more awareness. The total antioxidant capability, the activities/concentration of catalase, glutathione reductase, and the concentration of reduced-glutathione in the liver and spleen were increased in broilers (Arbor Acre) fed diets with *G. lucidum* sporoderm-broken spores (SSGL) at 200 mg/kg diet [63]. However, the mechanism by which SSGL promotes the antioxidant defense system is still unclear. *G. lucidum* triterpenoids reduce the cadmium content of the spleen, enhance the antioxidant enzyme activity, and reduce the expression of Malondialdehyde (MDA), which is a marker of oxidative stress in cadmium-induced broilers [111]. The antioxidative function of mushrooms such as *A. bisporus* is mainly due to phenolic compounds and polysaccharides [112,113]. *A. bisporus* stipes are reported to have more polysaccharides, phenolic compounds, and antioxidant capacity [14]. Aflatoxins are toxic and carcinogenic metabolites of *Aspergillus flavus*, *A. parasiticus*, and others [114]. The harmful effect of aflatoxin could be closely linked with the generation of reactive oxygen species, such as superoxide, hydrogen peroxide (H_2_O_2_), and hydroxyl [115]. Aflatoxicosis in poultry primarily affects the liver, which can adversely affect production. The levels of MDA, H_2_O_2_, and lipopolysaccharide in the liver of broilers fed *Ganoderma lucidum* (200 mg/kg diet) after being exposed to Aflatoxin B1 (AFB1) were markedly decreased [63]. In general, diets supplemented with medicinal mushrooms had better antioxidant effects.

## 8. Influence of Different Species of Mushrooms on the Gut Health of Birds

Gut microflora plays a major role in host nutrition, health, and growth performance [116] by interacting with the host gut system utilization and development. The interaction is multifaceted, and depending on the type and activity of the gut microflora, it can positively or negatively affect the growth and health of birds. Whenever pathogens attach to the mucosa, gut integrity and function are severely affected [117], and the immune system is challenged. Furthermore, it is generally accepted that gut microflora is a nutritional ‘‘burden” in fast-growing broiler chickens because an active microflora component may have an increased energy requirement for maintenance, thereby reducing the efficiency of nutrient utilization [118]. Adedokun and Olojede [119] suggested that an ideal flora should promote the absorption of nutrients during the digestive process whilst also ensuring that the host can mount an effective immune response during a pathogenic challenge. Mushroom supplementation in diets results in an alteration in the population of gut bacteria. The increase in the population of beneficial bacteria such as Bifidobacteria and Lactobacilli improves production parameters and increases chicken resistance to enteric infections and colonization by pathogenic bacteria such as salmonella [1,28,65,66,80].

Table 5 shows the influence of different species of mushrooms on the gut microbiota of birds.

## 9. Meat Quality

Oxidative stress in poultry can affect the meat quality of birds. Oxidative stress is associated with the development of the wooden breast disorder through the accumulation of reactive oxygen species (ROS) [120]. Since there is an increase in awareness of the nutritional quality of poultry meat consumed by consumers [121], there is a need to improve the meat quality. The oxidative status of animals and their products can be improved using antioxidants [122]. However, since the conventional use of synthetic antioxidants like hydroxyanisole and butylated hydroxytoluene may pose a public health hazard, there is a need to find natural antioxidant products that can help improve the antioxidant capacity of chickens and their products. It has been established that mushrooms possess active biological components that have antioxidant functions. Therefore, incorporating mushrooms into chickens’ diets can improve their antioxidant capacity and meat quality [18]. One of the meat quality indicators is pH, as its rate of decline is associated with meat tenderness. Refs. [45,83] noted that the inclusion of *Flammulina velutipes* stem residue in the broilers’ diet increased the pH of the meat. The loss of soluble nutrients, which can lead to poor meat quality, could be increased with a lower pH and water holding capacity [123]. Lower pH during post-mortem can increase the acidity in the muscle. The acidic environment can cause protein denaturation, thereby reducing the water-holding capacity and leading to drip loss of soluble nutrients. The inclusion of *Pleurotus eryngii* stalk residue increases the pH and water-holding capacity of the broiler’s meat [95].

Antioxidant-protective activity inhibits peroxidation of lipids, which is also responsible for meat quality deterioration, affecting flavor, texture, color, and nutritional value [1]. Meat color is one of the determining factors for customer preferences in buying poultry meat products. Altop et al. [14] noted that the dietary supplementation of *A. bisporus* cap at 20 g/kg inclusion in Ross 308 broiler increases the lightness of chicken thigh and water-holding capacity. Similarly, Vargas-Sánchez et al. [71] reported an increase in the meat quality parameters and antioxidant stability of quail breast meat because of dietary mushroom supplementation. Water loss is of commercial significance because it can generate economic losses when it exceeds 10.0%. Vargas-Sánchez et al. [71] reported that at day 15, the dietary *P. ostreatus* supplementation led to decreased water loss after cooking (8.0%), reduced the texture (10.0%), and increased the water-holding capacity (7.1%). However, it was reported that the taste quality of broiler meat fed with mushrooms was lower compared with the rest of the treatments. Camay [4] reported that the texture quality of broiler meat fed with 5 g *P. ostreatus* per kg of feed showed the lowest sensory rating, and it varies with the inclusion rate (10 g and 25 g). Similarly, the lowest general acceptability rating was observed in the broilers fed 5 g *P. ostreatus*. The oral administration of *Pleurotus ostreatus* at 4000 (mg/L) in water improved the meat quality of Cobb broilers via the improvement of oxidative stability of meat samples through the reduction in thiobarbituric acid reactive substances (TBARs) and increase in the glutathione peroxidase. The sequence of these processes positively stabilizes the meat tissue against lipid oxidation, thereby prolonging the quality of meat and its shelf life [124].

## 10. Conclusions

In conclusion, the use of mushrooms as a growth promoter in poultry is a major stride toward finding alternatives to antibiotics. Mushrooms have been effective in improving the birds’ growth performance, improving the feed conversion ratio, and increasing body weight gain. Thus, there is still a need to determine at what inclusion level a specific type of mushroom would confer a beneficial effect on the birds. The mechanism of action of mushroom-based supplements is poorly understood. It is important to understand the specific bioactive compounds responsible for the observed effects. Since there are many bioactive compounds present in different species of mushrooms, the isolation of these compounds is required for a better understanding of their role. Most of the studies on the egg quality of birds focused more on table eggs. However, there is little research on the influence of mushrooms on breeder eggs. Further research is needed on the in ovo application of mushrooms on hatchability and post-hatch performance. There is still a need to understand the best extraction methods that will yield the bioactive component well without compromising their efficacy. Pilot and large-scale studies involving mushroom-based supplements are still needed. Though the application of medicinal mushrooms in chicken feed generates positive results indicating their potential for antibiotic replacement, improved immunity, health, and growth enhancers, their acceptance and use in the mainstream poultry industry is limited.

## Figures and Tables

**Table 1:** Bioactive components of mushrooms and their functions in poultry.

S/N	Mushroom	Extraction Solvent	Bioactive Compounds	Function	References
1	*Agaricus bisporus*	Water, Ethanol, Methanol	Fucogalactan, Ergothioneine Phenols -Polysaccharides -Selenium -monosaccharide	inhibit colonization of pathogenic microbial flora in the intestines, Antioxidants	[1,28,29,30]
2	*Agaricus brasiliensis*	Chloroform, Methanol	Fucogalactan	Antibacterial	[37]
3	*Cordyceps militaris*	Methanol, Hot water extract	Glucose, D-mannitol, 3,4-Oisopropylidene- D-mannitol, Cordycepin (3′-deoxyadenosine), ascorbic acid	Antibacterial Antimicrobial, antioxidant and antitumor	[18,31]
4	*Pleurotus pulmonarius*	Water, ethanol, 95% Acetone	Glucan	Antibacterial	[38]
5	*Termitomyces* *albuminosus* (*Berk.*)	80% ethanol, water	Crude polysaccharide	Antibacterial	[39]
6	*Pleurotus sajor-caju* (Fr.) Singer	chloroform-methanol (2:1, *v*/*v*), water and ethanol	β-D-Glucan	Anti-inflammatory, antibacterial Properties	[40]
7	*Flammulina velutipes*	-Ethanol	Phenol	Antioxidant	[41]
8	*Pleurotus eryngii*	Water extract	-Phenol -Crude Polysaccharide	Antibacterial	[42]
9	*Tricholosporum goniospermum*	n-hexane, ethyl acetate and methanol extracts	phenolic compounds: gallic acid, catechin, epicatechin and resveratrol	Antioxidants	[32]
10	*Pleurotus djamor*	Methanol	Phenols Flavonoids	Antioxidants	[43]

**Table 2:** Effect of different species of mushrooms on growth performance.

S/N	Mushroom	Part/Preparation Form	Poultry	Inclusion Level	Effect on Performance	References
1	*Agaricus bisporus*	Whole mushroom/powder	Turkey	Feed-10 g/kg and 20 g/kg	- No effect on BW, WG and F:G at 28 days; - At 56 and 70 days, supplementation at 20 g/kg had better BW and WG.	[28]
2	*A. bisporus*	Whole mushroom/powder	Broiler (Ross 308)	Feed-10 g/kg and 20 g/kg	- No effect on FI, BW and WG up to 28 days of age; At d 42 of age, the 0.2% group had better BW and WG.	[1]
3	*A. bisporus*	Whole mushroom/powder	Broiler (Ross 308)	Feed-0.5, 1.0, 1.5, and 2.0 g/kg	Supplementation negatively affects feed intake	[57]
4	*A. bisporus*	Whole mushroom/powder	Broiler (Ross 308)	Feed-0.05, 0.1%, 0.2%, and 0.3%	- Improved daily feed intake in the group fed 0.1%; - Lowest FCR observed in 0.05% at the starter phase; - No effect on carcass traits.	[29]
5	*A. bisporus*	Whole mushroom	Broiler (Ross 308)	Feed-10 g/kg and 20 g/kg	- Broilers fed with 10 g/kg gained 2% higher body mass	[58]
6	*A. bisporus*	Stalk, Cap	Broiler (Ross 308)	Feed-10 g/kg stalk and 10 g/kg cap 20 g/kg stalk and 20 g/kg cap	- Chickens receiving A. bisporus diets had better FCR at 0–21 days; - BWG was decreased with the dietary inclusion of mushroom cap; - the FI of broilers decreased; - The best FCR was obtained by the dietary inclusion of mushroom stalk.	[14]
7	*Agaricus brasiliensis*	Whole mushroom/powder	Broiler (Ross 308)	Feed-1.6 and 2.0 g/kg	- Inclusion at 1.6 g/kg improved BWG and F:G ratio; - No effect on carcass traits.	[45]
8	*Cordyceps sinensis*		Broiler (Ross 308)	Feed-300 and 600 mg/kg	- Improved BWG with 600 mg/kg supplementation	[59]
9	*Flammulina velutipes*	Mushroom waste stem/powder	Broiler (Arbor Acre)	Feed-0.1, 0.2%	- ADFI, ADG, and FCR were not affected at the starter (1–21 days) and finishing (22–42 days) phase	[41]
10	*Flammulina velutipes*	Mushroom waste stem/powder	Pullet (ISA Brown)	Feed-0.2%, 0.4%, and 0.6%	- DWG and FCR were not affected; - Improved final LW in 0.4 and 0.6% groups.	[60]
11	*Ganoderma* *Lucidum*	sporoderm- broken spores	Broiler (Arbor Acre)	Feed 100, 200, and 500 mg/kg	- Increased ADFI and ADG and decreased F:G	[61]
12	*Ganoderma applanatum*	Fruit body/Aqueous extract	Eimeria Challenged broiler	100 g/500 mL extract	- Improved weight gain and reduced feed conversion ratio	[62]
13	*Ganoderma lucidum*	sporoderm- broken spores	Broiler exposed to low-level aflatoxin B1 (Arbor Acre)	Feed 200 mg/kg	Improved growth performance and reduced oxidative stress and immunosuppression of broilers	[63]
14	*G. lucidum*	fruiting bodies	Broiler (Ross 308)	Water-1 g/L	- No effect on growth performance	[64]
15	*G. lucidum*	fruiting bodies	Lipopolysaccharide-Challenged Broilers (Ross 308)	Water-1 mL/L and 1.33 mL/L	- Decreased body weight at 21 days of age	[65]
16	*Ganoderma* sp.	mature fruiting bodies	Pullet (Lorman brown)	Feed-2.0, 1.0, and 0.5 g/kg	- Improved F:G ratio with 2.0 and 1.0 supplementation; - No effect on FI.	[53]
17	*Lentinus edodes*	Whole	Broilers	Feed-0.5, 1, 2, 3, and 4 g/kg	- The overall mean of the BWG was improved	[66]
18	*Lentinus edodes*	Whole mushroom	Quail	Feed-0.5, 1, and 2%	- Linear decrease in LW; - LWG linearly decreased with increasing doses; - FCR was negatively influenced.	[67]
19	*Pleurotus* *ostreatus*	Whole mushroom	Harco Black pullet	Feed-1 g/kg	- Improved weight gain and feed conversion ratio	[68]
20	*P. ostreatus*	Mushroom powder	Broiler (Ross 308)	Feed-2 g/kg	- Decreased body weight in the first 21 days of age; - Reduction of weight gain and feed intake at 1–42 days of age.	[69]
21	*P. ostreatus*	Whole mushroom/powder	Broiler (Ross 308)	Feed-0.1 and 0.2%.	- Decreased FCR; - Supplementation at 0.2% improved feed efficiency and weight gain during the starting and growing phase. However, improvement was not sustained to the slaughter age	[2]
22	*P. ostreatus*	Mushroom waste stem/powder	Broiler (Ross 308)	Feed-1% and 2%	- Increased FI and BWG with 1% supplementation; - FCR was lower with 1% than 2%; - No effect on BWG and FI with 2% supplementation.	[70]
23	*P. ostreatus*	Mushroom waste stem/powder	Broiler	Feed-5, 10, 15, and 20 g/kg	- Decreased in body weights of broiler chickens 6 days after feeding with mushroom supplements; - improved the body weights as the broilers grow from 10 days to 28 days;	[4]
24	*P. ostreatus*	Whole mushroom	Quail	Feed-10 and 20 g/kg	- BWG, FI, FCR, and FE were not influenced	[71]
25	*Termitomyces microcarpus*	Whole mushroom/powder	Broiler	Feed-0.2%.	Supplementation resulted in poor bird performance	[72]

ADFI—Average Daily Feed Intake, FI—Feed Intake, BWG—body weight gain, FCR—feed conversion ratio, LWG—Live Weight Gain, LW—Live Weight, ADG—Average Daily Gain, F:G—feed-to-gain ratio, BW—body weight, WG—weight gain.

**Table 3:** Effects of different mushroom species on cholesterolemic properties in poultry.

S/N	Mushroom	Part/Form	Poultry	Effects	References
1	*Flammulina velutipes*	Stem waste	Broiler chickens (Arbor Acre)	Lower TC and HDL	[41]
2	*Pleurotus ostreatus*	Mushroom	Japanese quails	Lower TC, TG, and LDL Increase HDL	[92]
3	*Pleurotus ostreatus*	Mushroom powder	Broiler (Ross 308)	Lower HDL cholesterol	[93]
4	*Cordyceps militaris*	Spent mushroom substrate	Laying hens (Isa Brown)	Lower TC, LDL, and TG	[94]
5	*Pleurotus eryngii*	Stalk residue	Laying hens (Hendrix)	Lower serum TC and TG	[95]
6	*Pleurotus ostreatus*	In water and feed	Cockerel	Improve blood lipid profile	[96]
7	*Agaricus bisporus*	Mushroom powder	Broiler (Ross 308)	Reduced serum TG and VLDL	[57]
8	*Lentinula edodes*	Mushroom powder	Laying hens (Tetran Brown)	Reduce cholesterol concentration of egg yolk	[84]
9	*Pleurotus eryngii*	Stalk residue	Laying hen (Hendrix)	Lower cholesterol in egg	[95]

TC: total cholesterol; TG: triglycerides; HDL: High-Density Lipoprotein; LDL: Low-Density Lipoprotein; VLDL: Very Low-Density Lipoprotein.

**Table 4:** Effects of mushrooms on the immune response of birds.

S/N	Mushroom	Poultry	Inclusion Rate/Mode	Duration	Effect	References
1	*Agaricus bisporus*	Broiler (Ross 308)	Whole mushroom- 5, 10, 20, and 30 g/kg—Feed	45	- no effect on antibody titers against NDV - supplementation with 30 g/kg in diet could induce favorable effects on the humoral immune responses of broilers without compromising performance indices.	[29]
2	*Cordyceps militaris*	White roman chicken	Intramuscular injection—0.5 mL, 8.0 mg/mL, 4 mg/mL, and 2.0 mg/mL		Lymphocyte proliferation, enhanced serum antibody titer, and improved serum interferon-gamma and interleukin-4 concentrations	[81]
3	*Cordyceps militaris*	Broiler (Ross 308)	Hot water extraction; 2 g/L in water	35	It exerts an immunomodulatory function and inhibits inflammation-related gene expression in response to vaccination and LPS stimulation in broilers	[18]
4	*Flammulina velutipes*	10 weeks old Pullet (ISA Brown)	Stem; 2%, 4%, and 6%—Feed	42	- The best responses for antibody titers were found in pullets fed 6% - The highest concentrations for immunoglobulin parameters were observed in both 4% and 6%	[60]
5	*Flammulina velutipes*	Broiler (Arbor Acre)	Stem waste; 1% and 2%	42	- A significantly higher antibody response against NDV and IBD - Antibody titers against NDV were found to be higher in the 2% MW	[41]
6	*Ganoderma lucidum*	Broiler (Arbor Acre)	sporoderm- broken spores; 100, 200, and 500 mg/kg—Feed	44	Supplementation has ameliorative effects on immune function	[61]
7	*Pleurotus florida*	Broiler	Isolated β-glucan; 15 mg and 30 mg—feed	20	*P. florida* has an immunostimulatory effect due to the presence of beta-glucan	[98]
8	*Pleurotus osteratus*	Broiler (Ross 308)	Stem waste; 1 and 2%—Feed	42	The optimal responses for antibody titer was found in broilers fed 1% The highest concentrations for immunoglobulin parameters was observed in the 1% OMW group.	[70]
9	*Pleurotus osteratus*	Broiler (Ross 308)	Whole mushroom-10 and 20 g/kg—Feed	42	promotes antibody titer production against ND and influenza viruses	[2]
10	*SCRO* *	Natural coccidiosis-infected broilers (Ross)	Myceliated Shiitake (5, 10%), Cordyceps (5, 10%), Reishi (5, 10%), and Oyster mushroom (5, 10%)	49	reduced lymphocyte percentages in 5% reishi reduced heterophils in 5% oyster	[15]
11	*Tremella fuciformis*	*E. tenella*-infected broiler	Whole—1 g/kg	34	- higher immune response at 21 d post-infection; - it enhanced cellular and humoral immune responses in E. tenella-infected chickens.	[66]

* SCRO—Shiitake, cordyceps, reishi, oyster Mushrooms; ND—Newcastle disease; NDV—Newcastle disease virus.

**Table 5:** The influence of different species of mushrooms on the gut microbiota of birds.

S/N	Mushroom	Poultry	Inclusion Rate	Bacteria	Site	Effect on Bacteria Population	References
1	*Agaricus bisporus*	Turkey	Intact mushroom Feed-10 g, 20 g/kg	- lactobacilli - *E. coli* - lactobacilli	- Ileum - Ileum - cecum	- Increased - Decreased - Increased	[28]
2	*Lentinus edodes*	Salmonella-infected molting Hens (Single Comb White Leghorn)	Mycelium	- Salmonella - Salmonella	- Ceca - Crop	- Decreased - Decreased	[80]
3	*Lentinus edodes*	Broilers naturally infected with Mycoplasma gallisepticum	Whole	- *Bacteroides* spp., enterococci, *E. coli* - Bifidobacteria - Lactobacilli	- Ileum - Cecum - Rectum	- Decreased - Increased - Increased	[66]
4	*Agaricus bisporus*	Broiler (Ross 308)	Intact mushroom Feed-10 g, 20 g/kg	- *lactobacilli* spp. - *E. coli*: *Lactobacilli* spp. - Total anaerobes, Total aerobes, *Clostridia* spp., *Bifidobacteria* spp., *E. coli*, *Bacteroides* spp. Enterococci - *Lactobacilli* spp. - *Bifidobacteria* spp. *E. coli*: *Lactobacilli* spp. -Total aerobes, total anaerobes, *Clostridia* spp., *E. coli*, *Bacteroides* spp. and Enterococci.	- Ileum - Ileum - Ileum - Cecum - Cecum - Cecum - Cecum	- increased 20 g/kg - Decreased Unaffected - Increased - Increased - Decreased Unaffected	[1]
5	*Ganoderma Lucidum*	Lipopolysaccharide-Challenged Broilers (Ross 308)	Water-1.33 mL/L	phylum Firmicutes phylum Bacteroidetes Barnesiella and Lactobacillus	- Feacal - Feacal - Feacal	- Increased - Increased - Increased	[65]
6	*Agaricus bisporus*	Broiler (Ross 308)	Feed-10 g, 20 g/kg	- *Lactobacilli* spp.	-Ileum and Cecum	-Increased	[1]
7	*Agaricus bisporus*	Broiler (Ross 308)	Feed-5 g, 10 g, 20 g, 30 g	- *Lactobacilli* spp. - *Escherichia coli*	-Ileum	-Increased -Decreased	[29]
8	*Pleurotus eryngii*	Broiler (Ross 308)	Feed-5%	- Firmicutes: Bacteroidetes - Bacteroidetes: Verrucomicrobia Firmicutes/Bacteroidetes Epsilonbacteria and Bacteroidetes	- Cecum - Cecum - Ileum - Ileum	Decreased - Increased Decreased - Increased	[42]
9	*Lentinus edodes*	Quail	Feed-0.5, 1 and 2%	Lactobacillus *E. coli*	- Small intestine	Decreased - Increased	[67]

## Data Availability

Not applicable.
